# Effectiveness of social distancing measures and lockdowns for reducing transmission of COVID-19 in non-healthcare, community-based settings

**DOI:** 10.1098/rsta.2023.0132

**Published:** 2023-10-09

**Authors:** Caitriona Murphy, Wey Wen Lim, Cathal Mills, Jessica Y. Wong, Dongxuan Chen, Yanmy Xie, Mingwei Li, Susan Gould, Hualei Xin, Justin K. Cheung, Samir Bhatt, Benjamin J. Cowling, Christl A. Donnelly

**Affiliations:** ^1^ World Health Organization Collaborating Centre for Infectious Disease Epidemiology and Control, School of Public Health, Li Ka Shing Faculty of Medicine, The University of Hong Kong, Hong Kong, People's Republic of China; ^2^ Department of Statistics, University of Oxford, Oxford, UK; ^3^ Laboratory of Data Discovery for Health, Hong Kong Science and Technology Park, New Territories, Hong Kong, People's Republic of China; ^4^ Department of Clinical Sciences, Liverpool School of Tropical Medicine, Liverpool, UK; ^5^ Tropical and Infectious Disease Unit, Liverpool University Hospitals NHS Foundation Trust, Liverpool, UK; ^6^ Section of Epidemiology, Department of Public Health, University of Copenhagen, Kobenhavn, Denmark; ^7^ MRC Centre for Global Infectious Disease Analysis, Department of Infectious Disease Epidemiology, School of Public Health, Faculty of Medicine, Imperial College London, London, UK; ^8^ Pandemic Sciences Institute, University of Oxford, Oxford, UK

**Keywords:** transmission, social distancing, lockdown, SARS-CoV-2, schools, care homes

## Abstract

Social distancing measures (SDMs) are community-level interventions that aim to reduce person-to-person contacts in the community. SDMs were a major part of the responses first to contain, then to mitigate, the spread of SARS-CoV-2 in the community. Common SDMs included limiting the size of gatherings, closing schools and/or workplaces, implementing work-from-home arrangements, or more stringent restrictions such as lockdowns. This systematic review summarized the evidence for the effectiveness of nine SDMs. Almost all of the studies included were observational in nature, which meant that there were intrinsic risks of bias that could have been avoided were conditions randomly assigned to study participants. There were no instances where only one form of SDM had been in place in a particular setting during the study period, making it challenging to estimate the separate effect of each intervention. The more stringent SDMs such as stay-at-home orders, restrictions on mass gatherings and closures were estimated to be most effective at reducing SARS-CoV-2 transmission. Most studies included in this review suggested that combinations of SDMs successfully slowed or even stopped SARS-CoV-2 transmission in the community. However, individual effects and optimal combinations of interventions, as well as the optimal timing for particular measures, require further investigation.

This article is part of the theme issue 'The effectiveness of non-pharmaceutical interventions on the COVID-19 pandemic: the evidence'.

## 1. Introduction

Social distancing measures (SDMs) are interventions applied to individuals in the community that aim to reduce transmission by reducing person-to-person contacts or the chance of transmission when contact occurs, regardless of their infection or exposure status. The use of SDMs as a means to reduce community transmission of infectious diseases dates back to the 1918 influenza pandemic when school closures and restrictions on mass gatherings were implemented in the United States (US)—a policy decision that was later estimated to have saved thousands of lives [1,2]. School closures were also implemented during the 2009 influenza A(H1N1) pandemic. However, as infections were generally of mild-to-moderate severity and antiviral treatments and vaccines became quickly available, SDMs were implemented for less than a year. These SDMs were also less restrictive and measures to restrict human mobility more generally were not implemented. During the COVID-19 pandemic, restrictions on mass gatherings, school closures, business closures and restrictions on human mobility (both within and across national borders) were implemented in most countries and in many cases for prolonged periods of time.

SDMs may be applied to specific community settings where there is thought to be a higher risk of disease transmission or a higher impact of outbreaks than the general community. With the help of rigorous contact tracing in some locations, it was identified early in the COVID-19 pandemic that clusters of cases were occurring in settings that involved close interpersonal interactions or industries that require their employees to work directly with clients or the public [3,4]. Social and dining activities that occur in restaurants, bars and weddings were associated with more secondary cases than households for individuals of the same age [5]. Many social settings and environments where personal care services are delivered with the removal of face masks may be at higher risk of disease transmission in those settings, especially when they occur in enclosed spaces. As such, SDMs that limited group sizes for dine-in restaurants, reduced the capacity of venues or limited opening hours were implemented. Outbreaks in long-term care facilities were particularly concerning because infections in frail older adults were often more severe than those in younger individuals. SDMs in care homes during the COVID-19 pandemic generally took the form of cohorting residents and staff or restricting visitors, where sick or exposed residents were grouped together and/or dedicated staff were assigned to only work within those groups. This is challenging as care homes often rely on agencies to provide staff on an on-call basis due to well-documented pre-existing staffing challenges [6]. In 2009, it was estimated that up to 60% of nursing homes in the US relied on agency staff [7]. This reliance meant staff members would commonly work in multiple care homes, which contributed to the introduction of infections in some care homes during the pandemic. A study carried out in the US in 2020 found that the risk of infection in nursing homes increased ninefold if the homes hired staff through agencies [8].

Lockdowns, also known as stay-at-home orders, were the most restrictive SDM where the majority of the population were required to stay-at-home with exceptions granted only for exercise and essential shopping. The stringency of this measure varied across the world due to the need to balance preventing transmission that could lead to numerous hospitalizations and deaths while also avoiding large contractions of the economy or the breakdown of essential services. Therefore, some stay-at-home orders were targeted at some segments of the community rather than community-wide, for example, allowing construction sites or factories to remain open. Here, we focused on reviewing the effect of SDMs in community settings, including those applied in high-risk settings.

## 2. Methodology

Individual search terms and systematic literature searches were performed to obtain studies reporting the effectiveness of nine specific SDMs (school closures; school measures; workplace closures; workplace measures; catering, fitness and personal care service measures; care home measures; restrictions on mass gatherings; physical distancing and stay-at-home orders) on the transmission of SARS-CoV-2 in the community (electronic supplementary material,appendix B).

Database searches were carried out in Web of Science and Scopus from 1 January 2020 to 1 December 2022. All study designs were considered for inclusion in this review. However, if there were more than 10 observational studies or studies of higher quality of evidence such as randomized controlled trials or quasi-experimental studies, simulation studies (defined as modelled scenarios without fitting to any observed data) were excluded. Throughout this review, the term ‘ecological study’ refers to the investigation of an association using population-level rather than individual-level data, and may, therefore, be vulnerable to ecological fallacy [9]. Studies that use statistical or mathematical modelling methods that were fitted to observed data will be referred to in this review as ‘modelling studies’ while studies that only simulate hypothetical epidemics based on parameter estimates or assumptions will be referred to as ‘simulation studies’. Preprints were excluded from this review. Broad Google searches were also conducted to find any existing systematic reviews, and relevant studies included in these reviews were also included here.

We included in this review quantitative studies that estimated the effect of SDMs implemented in the community setting that were aimed at reducing SARS-CoV-2 transmission or disease severity by reducing person-to-person contacts or making such contacts safer. Community settings were defined as non-healthcare settings where medical care by health professionals is not usually delivered, including homes, schools, workplaces and long-term care facilities in the community. Interventions designed to reduce transmission in the community through other mechanisms, such as improved ventilation or face masks, are not considered in this review.

The titles, abstracts and full texts of search results were screened by two reviewers. Data were extracted from included studies, and the quality of the evidence was assessed using the Grading of Recommendations Assessment, Development and Evaluation (GRADE) framework—a tool for evaluating the evidence available for an intervention based on eight criteria, four of which are based on the risk of biased estimates due to study design or measurement errors [10,11]. In this framework, the certainty or quality of evidence is categorized into four levels: very low, low, moderate or high. The quality of evidence from randomized trials is initially rated as high, and evidence from observational studies is initially rated as low. This initial rating is then penalized when there are potential risks of bias (e.g. selection or misclassification bias), inconsistencies of findings with published literature, indirectness of reported outcomes compared with true outcomes (e.g. the use of non-specific outcomes), imprecise measurement of exposure or outcomes and the likelihood of publication bias; or upgraded if the reported effect size is large despite plausible residual confounding that may reduce or nullify the effect, and has an appreciable dose–response relationship between intervention and outcomes [11]. Thus, findings from well-conducted randomized controlled trials would generally be considered high-quality evidence with this tool. By contrast, observational studies are often classified as either low or very-low quality.

Data extraction and GRADE assessments were conducted using Microsoft Excel, and further analyses of extracted data were conducted using R v. 4.2.1 (R Foundation for Statistical Computing, Vienna, Austria).

## 3. Results

**Figure 1.**
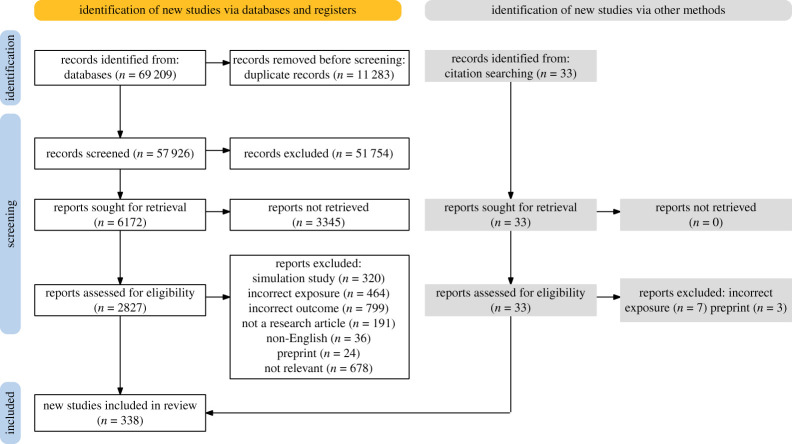
Combined flow diagram for the review of nine social distancing measures: (1) school closures (SC); (2) school measures (SM); (3) workplace closures (WC); (4) workplace measures (WM); (5) catering, fitness and personal care service measures (CFP); (6) care home measures (CH); (7) restrictions on mass gatherings (RMG); (8) physical distancing (PD) and (9) stay-at-home orders (SAH).

The nine systematic reviews included 338 studies, among which 48 reported effectiveness estimates for more than one SDM (figure 1, electronic supplementary material, appendix A, Table S3) [12–59]. Most studies analysed population-level data and examined SARS-CoV-2 transmission and/or COVID-19 mortality and morbidity (including hospitalizations and deaths) in the presence or absence of the intervention. The main reasons studies were excluded were that the intervention was not evaluated in a community setting or the outcome was unrelated to the effect of the intervention on SARS-CoV-2 transmission (figure 1). As most of the evidence identified in this review came from observational studies, the quality or certainty of the evidence was mainly rated as low or very low for most studies (electronic supplementary material, appendix A) based on the GRADE framework, indicating that the true effect may differ from the estimated effect. However, with over 300 studies included in this study, patterns indicative of the effectiveness of SDMs have emerged. When modelling studies or simulation studies were included due to a lack of randomized or observational studies, their quality of evidence was not assessed because the GRADE framework was not designed for their assessment. In general, simulation studies that did not fit models to data would typically be considered to provide a lower quality of evidence than observational studies or modelling studies that did fit mathematical models to epidemic curves.

Owing to the high volume of papers and concerns over the quality of evidence presented in some studies, we highlight here studies that were conducted more thoroughly (attempted estimating a causal relationship while adjusting for confounders and quantifying uncertainty) and examined whether other studies supported their findings. Details of the included studies are provided in electronic supplementary material, appendix A, and the visualization of their study period and reported effects are included in electronic supplementary material, appendix C.

### (a) Stay-at-home orders

During the COVID-19 pandemic, stay-at-home orders were also referred to as lockdowns, shelter-in-place, mandatory control orders or in some locations as circuit-breaking measures. Italy was the first European country to implement stay-at-home orders on 9 March 2020, lasting over 60 days. As infections spread, the UK also announced a lockdown on 23 March 2020 and began a phased reopening by mid-May that year. Here, we included 151 studies estimating the effectiveness of stay-at-home orders (electronic supplementary material, appendix A, Table S13), 119 of which found a substantial benefit resulting in a reduction of the reproduction number (Rt) [16,23,33,35,38,45,48,60–97], incidence of SARS-CoV-2 infection [29,50,52,98–129] and mortality [107,116,130–143].

Among the studies that reported a relative reduction in Rt, most estimated substantial reductions of around 50%, although there was a wide range of effects (6–81%). These studies had different study designs, populations and definitions for stay-at-home orders. They were mainly carried out at a national scale within the first year of the pandemic (electronic supplementary material, appendix C). Definitions differed in stringency, where a stay-at-home order may include lockdown-type measures such as restricting internal travel and imposing limitations on gatherings versus the most stringent where individuals were unable to leave their homes for anything other than exercise or essential shopping. A modelling study that estimated the effects of 17 non-pharmaceutical interventions across two waves in seven European countries estimated that a lockdown (banning all gatherings and closing all non-essential businesses) reduced the Rt by 52% (95% CrI: 47%, 56%) [45]. Two studies in the US in 2020 found similar reductions, estimating a reduction of Rt by 51% (95% CI: 46%, 57%) after stay-at-home orders were implemented in some states [38,80]. However, another study carried out on a multi-national scale in early 2020 concluded that stay-at-home orders had a relatively small additional effect (on top of business closures, school closures and gathering restrictions that were already in place), reducing the Rt by 13% (95% prediction interval (PI): 5%, 31%) [16]. A study in Europe in 2020 also estimated a smaller additional effect of lockdowns when implemented on top of other measures [28].

Studies that estimated the impact of stay-at-home orders on COVID-19 incidence varied across settings. A study in Australia from 2020 to 2021 estimated that the lockdown in Victoria decreased the incidence of COVID-19 two weeks after its implementation (incidence rate ratio (IRR): 0.88; 95% CI: 0.86, 0.91) [113]. In comparison, a multi-national analysis that looked at 210 countries in early 2020 found that stay-at-home orders reduced the incidence of COVID-19 by 11.2% [52]. Three studies did not find a significant association between stay-at-home orders and COVID-19 cases [14,28,144]. However, the effectiveness of stay-at-home measures on reducing mortality was mixed, with 16 studies [107,116,130–141] reporting reductions, and nine studies reporting no significant associations [19,28,30,47,144–148]. Nevertheless, one study in Europe [28] and another in the US [144] concluded that social distancing behaviours had already changed substantially before stay-at-home orders were implemented in early 2020, and therefore little additional benefit was observed after the stay-at-home orders were issued.

### (b) School measures and closures

Historically, children have played an important role in influenza transmission due to their susceptibility to influenza virus infection and prolonged viral shedding, which facilitates transmission to family members and, by extension, the community. Based on this prior understanding, schools in many countries were proactively closed during the pandemic. In some locations, however, interventions to reduce within-school transmission were used as an alternative to complete closures of schools. These include rotating school schedules (e.g. children from different grades may be in school at different times), reducing the number of consecutive school days, physical distancing or limiting classroom capacities. Some classes were dismissed early to avoid having meals together or if a case was identified in that class.

Eighteen included studies estimated the effectiveness of school measures (not including closures) to reduce the impact of COVID-19. Six were observational studies [149–154] and 12 were simulation studies [155–166]. Most studies were carried out in 2020 at a sub-national scale (electronic supplementary material, appendix C). Seven of these studies estimated the effect of school measures in combination with the mandatory or recommended use of face masks [149,151–153,155,157,160].

Four of the six observational studies assessed individual schools between the end of 2020 and early 2021. Three did not quantify the effect but observed minimal transmission in schools with SDMs and universal masking interventions in place despite substantial community transmission [150,151,153]. The remaining study that examined the effect of multiple distancing measures in 36 schools in Italy (including limited capacity, distanced student desks and minimized crowding and entry and exits) observed that the overall secondary transmission rate was 3.8%, although there was no comparator without the interventions in place [152]. Two other observational studies had similar findings [150,154]. One that used data from 35 school outbreaks across 12 countries from 2020 to July 2021 [154] suggested that distancing and masking were both associated with a lower risk of SARS-CoV-2 infection in schools (adjusted odds ratio (aOR): 0.30, 95% CI: 0.25, 0.37) [154]. Another ecological study examining schools in North Carolina and Wisconsin, US, from 2020 to 2021 did not observe an increase in the secondary transmission rate in schools after distancing measures were relaxed, indicating they had no effect on transmission in these schools [149]. The remaining simulation studies found that school measures were associated with reductions in public health impacts of COVID-19, both in the schools [156–158,160,162–166] and the community [155,159,161].

For the review of school closures, 104 studies were included. All the studies were observational; about 89% estimated the impact of proactive school closures during the COVID-19 pandemic. The remaining 11% estimated the impact of reopening of schools. Forty-eight studies were conducted on a national scale, and 35 were on a multi-national scale. Over half of these studies showed strong evidence for the effectiveness of this intervention. Compared with the other individual interventions, school closures were examined for longer into the pandemic, with 29 studies assessing their effectiveness in 2021 (electronic supplementary material, appendix C).

Thirty-two studies estimated the effects of school closures on a country's community epidemics [13,17,19,23–26,28,31,34,36,38,39,41,42,44,46,51–54,56,58,167–175] and 26 studies estimated the impact of closures on students, staff or specific age groups in the population [40,176–200]. Ten studies estimated the relative reduction of Rt in the population [23,26,34,38,39,42,51,53, 170,171] with school closures implemented alone or in combination with other interventions. One study that attempted to discern the individual effect of school closures estimated they were responsible for reducing the Rt of SARS-CoV-2 in the US in the first half of 2020 by 37% (95% CI: 33%, 40%). This was followed by daycare closures (31%, 95% CI: 26%, 35%) [95]. In comparison, another study in the US carried out from January to May 2020 found school closures were associated with a 10% reduction in the daily Rt [38].

Several studies estimated the effect of proactive school closures internationally and estimated reductions in the incidence [13,25,28,34,54,58] and transmission [31,39,41,42,44] of SARS-CoV-2, and their associated hospitalization [46] and mortality [19,172–174]. The impact of school closures varied potentially due to differences in study populations, study period and the timing of implementation of school closures across settings. A study that estimated the independent contribution of school closures across 30 European countries from January to April 2020 reported that the IRRs for COVID-19 cases were estimated at 52% 22–28 days after school closures compared with a pre-intervention baseline). This estimate was 14% after more than 36 days of school closures [28], which indicated negative associations between school closures and the IRR for COVID-19 cases. These associations were unclear in six studies conducted on a multi-national scale in 2020 [17,24,36,52,56,175]. One study that looked at the general population across 90 countries did not observe statistically significant effects of school closures on daily confirmed COVID-19 cases during the first global wave of the pandemic but estimated significant reductions in the second and third waves [175].

Twelve studies examined the impact of reopening schools [21,201–211]. Nine showed an increasing trend in the number of daily new confirmed COVID-19 cases, growth rate or Rt of COVID-19. One study in South Carolina, US, estimated the Rt increased by 12.3% (95% CrI: 10.1%, 14.4%) after schools reopened at the end of August 2020 [21]. However, one study found that the reopening of schools did not immediately impact SARS-CoV-2 incidence, and an increase in incidence was not observed until 13 weeks after reopening [203]. The remaining four studies observed that reopening schools did not generate a substantial increase in transmission within the community when other interventions to prevent SARS-CoV-2 transmission were in place, including staff wearing masks, hand sanitation and limiting the in-person capacity in schools [198,208–210].

Sixteen studies also examined different school closure strategies [12,20,37,47,48,55,59,212–220]. For instance, two studies estimated that the delay of school closures in March 2020 was associated with more deaths across 50 states in the US [212,213]. Similarly, two more studies estimated the impact of the timing of the implementation of school closures [55,214]. One study in the US estimated that every additional day of delay from a county's first case until implementation of school closures, was associated with 1.5–2.4% higher cumulative COVID-19 deaths per capita (980–1972 deaths) for a county with median population and deaths per capita [214]. In Pakistan, a city that implemented complete school closures for 10 days saw a greater decline in incidence for the overall city population compared with a city that partially closed schools [217]. In the remaining 18 studies [22,27,36,45,49,50,221–232], the effects of school closures were estimated alongside other interventions, making it challenging to isolate the individual effect of school closure strategies.

### (c) Workplace measures and closures

Twelve observational studies were included in the review for workplace measures [17,21,41,55,233–240]. These were carried out at a national and sub-national scale, with nine studies conducted within the first year of the pandemic (electronic supplementary material, appendix C). Ten studies examined the effectiveness of workplace measures using population-level data [17,21,55,234,236–238,240–243]. Six studies assessed workplace mobility to explain the variation in the COVID-19 growth rate, case numbers or Rt [17,55,234,236,237,240] consistently showed that reduced workplace mobility was significantly associated with reduced SARS-CoV-2 incidence or transmission [234,236,237,240]. Some analyses were based on data from a single country [17,55], and others considered data from different countries [41,235].

There were two retrospective cohort studies that estimated the effectiveness of individual measures instead of a combination of measures at a population level [233,239]. Seven companies in Spain implemented a digital application in May 2020 that monitored workers in real-time to enable the quick identification and isolation of workers. Over a seven-month period, the proportion of symptomatic employees continuously decreased [233]. Another study on temperature screening implemented by 20 multi-national companies in February 2021 [239] found that the detection of COVID-19 cases using this measure alone was very rare, and approximately 2000 workers who were diagnosed with COVID-19 during the study period were not identified [239].

Thirty-seven studies were included in the review for workplace closures, 90% of which estimated effects in 2020. It is, therefore, unclear whether the levels of effectiveness would be similar for newer SARS-CoV-2 variants. All the included studies were observational, and they estimated the effect of workplace closures at a population level alongside other interventions. Most studies (92%) observed a beneficial effect of workplace closures alone or in combination with other interventions to reduce incidence [15,24,25,27,29,32,46,52,244–250] of COVID-19 and transmission [18,23,24,33,35,38,39,43,48,49,251] of SARS-CoV-2. A study that reported the examined effect of workplace closures alone estimated that non-essential workplace closures across 13 countries in Europe from March to May 2020 were estimated to reduce the change in deaths by 4% points (95% CI: 0.5, 7.4 pp). Another study based on county-level data in the US in early 2020, estimated that had SDMs such as non-essential business closures and stay-at-home orders been implemented a day earlier, the COVID-19 death rate could have been lowered by 1.9% [252]. However, a study using data from 10 countries (England, France, Germany, Iran, Italy, Netherlands, Spain, South Korea, Sweden and the US) that compared more stringent SDMs (mandatory business closures and stay-at-home orders) in England with less stringent policies in Sweden and South Korea in early 2020 did not observe additional effects with more stringent measures on the growth rate of cases in any country [14].

### (d) Catering, fitness and personal care service measures

Nine studies estimated the effectiveness of measures in catering, fitness and personal care service settings, including one randomized controlled trial [253], one cross-sectional study [254], three ecological studies [57,255,256] and four simulation studies [257–260]. One study examined fitness centres [253], and another looked at the reopening of theatres [260], while the remaining 10 studies estimated the effectiveness of SDMs in restaurants and bars. Six studies were conducted on a national or sub-national scale, while three simulation studies had an unclear setting or study period [258–260].

Catering measures appeared to be effective at reducing SARS-CoV-2 infection and Rt, although the effect varied by specific distancing measures. A study in Spain estimated that shortened bar and restaurant business hours and restricted outdoor seating capacity from August 2020 to January 2021 were associated with significant reductions in Rt of 0.14 and 0.11 respectively [57]. Similarly, in Norway, SARS-CoV-2 infection among bartenders and waiters had been reduced by 60% from 2.8 (95% CI: 2.0, 3.6) per 1000 workers to 1.1 (95% CI: 0.5, 1.6) per 1000 workers) four weeks after the implementation of a ban on serving alcohol in late 2020 to early 2021. The partial ban decreased infections among bartenders and waiters by 50% from 2.5 (95% CI: 1.5, 3.5) per 1000 workers to 1.3 (95% CI: 0.4, 2.2) per 1000 workers [254]. However, this was not supported by two observational studies that were both carried out in 2020 in Hong Kong and Tokyo, Japan [255,256]. The ban on dine-in services after 18.00 in restaurants in Hong Kong may not have influenced Rt after capacity reductions had already been considered [255]. Similarly, the randomized controlled trial carried out in Norway in 2020 suggested that there was no significant difference in laboratory-confirmed SARS-CoV-2 infections between the intervention (access to a fitness centre implementing prevention control measures) and control (no access to fitness centres) groups after 14 days. However, there were concerns in several aspects of the trial, such as the low incidence of COVID-19 during the trial period and the risk of transmission by exercising in groups, whether in a fitness centre or not [261].

### (e) Care home measures

Sixteen studies, including 11 epidemiological studies [53,262–271] and five simulation studies [272–276] examined the effect of SDMs on SARS-CoV-2 transmission in care homes or the effect of care home SDMs on population-level transmission. Nine of the 16 studies were carried out at a sub-national scale (electronic supplementary material, appendix C).

Two studies were chosen to discuss in depth as they both reported the effect of cohorting of staff and/or residents while taking confounding factors into consideration. One study on long-term care facilities in the south-west of France in early 2020 estimated that if staff were organized into smaller groups to work in different areas of the facility with no physical connection to other groups, there was a reduced risk of infection (odds ratio (OR): 0.19, 95% CI: 0.07, 0.48) [268]. This was not the case if residents were similarly compartmentalized (OR: 3.01, 95% CI: 0.51, 18.51) or if they were restricted to their rooms (OR: 1.67, 95% CI: 0.49, 5.76) [268]. However, the 95% confidence intervals for compartmentalizing residents were very wide owing to the small sample that reported they implemented this intervention (less than 20% of the 124 long-term care facilities examined). A second study carried out in the UK during mid-2020 reported that both the risk of infection in residents (aOR: 1.30, 95% CI: 1.23, 1.37) and staff (aOR: 1.20, 95% CI: 1.13, 1.29) were significantly higher in long-term care facilities in which staff often or always cared for both infected or uninfected residents compared with those that always cohorted staff [269].

Rigorous modelling, fitted to surveillance data in England, examined a different care home measure: restricting the number of visitors per nursing home resident [265]. It estimated the impact of reducing the contact rate between care home residents and the general population by 50% in 2020. However, compared with a baseline scenario with no reduction in contacts, the study did not find a substantial difference in care home deaths [265]. It was suggested that this may have been due to other routes of transmission into care homes at the time, such as patients being discharged from the hospital to care homes without being tested, indicating the importance of understanding the routes of transmission to increase the effectiveness of interventions and reduce the impact of COVID-19. This was supported by another simulation study in English nursing care homes [275].

Two population-based studies [264,266] ranked the US state-level restrictions by the stringency of their measures and compared COVID-19 incidence across states. Using data from just over 6800 nursing homes, one study estimated that the states with more stringent measures had an 11% reduction in the new cases in residents (IRR: 0.89, 95% CI: 0.83, 0.97) compared with states with less stringent restrictions [266]. Similarly, the risk of infection for those residing in assisted living communities was lower in states with more stringent measures [264]. In the first half of 2020, the US implemented a ban on visits to nursing homes to reduce contact with the community when the prevalence was high. This was estimated to reduce the weekly effective reproduction number of SARS-CoV-2 across 3035 US counties by 26% (95% CI: 23%, 29%) [53].

Other than cohorting and restricting visitors, staff working in multiple care homes was considered a large source of SARS-CoV-2 introductions and subsequent outbreaks [277]. The role of these connections in spreading COVID-19 cases was examined among nursing homes in the US in 2020 by analysing smartphone location data from 50 million smartphones over an 11-week period. Results indicated that if a nursing home adds one neighbour (a home with at least one shared contact) the expected number of COVID-19 cases increases by 15.2% and further suggests that 49% of cases in nursing home residents were attributed to staff transmitting SARS-CoV-2 across numerous homes [262].

### (f) Physical distancing and restriction of mass gatherings

Physical distancing is a measure taken by individuals to stay a recommended distance between one another (usually a minimum of 1 m) to limit transmission [278]. However, distancing oneself from others is also the key to many of our community-wide measures, and therefore, the term physical distancing also encapsulates measures including restrictions on mass gatherings, working from home, staying at home or adaptations to schools and workplaces [278]. Thirty-four studies were identified in the review of physical distancing (electronic supplementary material, appendix A) [13,18,26,29,32,33,42,45,51,56,279–302], 19 of which were observational studies. Most studies were carried out in 2020 and at a national scale, and 33 studies found physical distancing effective (electronic supplementary material, appendix C).

A rigorous modelling study that examined the individual effects of interventions in seven European countries from 2020 to 2021 found business closures were particularly effective with a 35% (95% CI: 29%, 41%) reduction in Rt while gastronomy and nightclubs were estimated to reduce the Rt by 12% (95% CI: 8%, 17%) each, and the closure of leisure centres, entertainment venues, zoos and museums had minimal effects. Considered collectively, banning all gatherings, including one-to-one meetings, had a large effect with a 26% (95% CI: 18%, 32%) reduction in Rt [45]. Seven other studies [26,29,33,42,56,283,286] attempted to estimate individual effects. The remaining studies examined the combined effects of different packages of measures due to the differing interventions included in the term physical distancing. For example, another study across 50 states in the US during the first five months of 2020 estimated social distancing was associated with a 15.4% daily reduction in COVID-19 cases where the SDMs included limits on the size of group gatherings, closures of public schools and non-essential business and stay-at-home orders [32]. Only one study assessed physical distancing at an individual level between students in schools. The study compared public schools in Massachusetts, US, that implemented greater than or equal to 6-feet distancing to those with greater than or equal to 3-feet distancing between students. However, likely due to a small sample size, it did not capture any additional effects that distancing by 6 feet may have [301].

If physical distancing measures had been implemented earlier many infections and deaths could have been avoided, two studies suggested [279,296]. One study in New York, US, estimated that if the interventions were implemented a week earlier, the total number of COVID-19 cases would have reduced by nearly 162 000 as of 31 May 2020. If there was a one-week delay, the total number of cases could have increased from 203 261 to 1 407 600 [279]. Cases and deaths were also estimated to reduce by 35.2% and 30.8% in Iran from January to September 2020 if physical distancing interventions (including school and border closures) and self-isolation were implemented a week earlier [296].

The effectiveness of restricting mass gatherings for reducing the impact of COVID-19 was examined in 28 studies [12,16,18,19,23,28,33–35,38,39,41,43–45,52,57,59,303–312], and 26 reported a substantial reduction in the impact of COVID-19. The most common outcome was the Rt [16,18,23,33,35,38,39,43,45,57,307–310] of SARS-CoV-2. Only two studies did not find statistically significant effects of the restriction on mass gathering on SARS-CoV-2 transmission [19,23]. The majority of studies were carried out in 2020 on a multi-national scale (electronic supplementary material, appendix C). Half of the studies (14) made use of the Oxford COVID-19 Government Response Tracker [16,18,19,33–35,39,44,59,303–305].

A comprehensive modelling study, using data from 41 countries, estimated the effects of different levels of stringency for restrictions on gatherings in the first half of 2020 [16]. The associated reduction in Rt for restricting gatherings to 1000 people or fewer was 23% (95% PI: 0%, 40%); limiting gatherings to 100 people or fewer was 34% (95% PI: 12%, 52%) and limiting gatherings to 10 people or fewer was 42% (95% PI: 17%, 60%) [16]. That the effectiveness of restricting mass gatherings increased as the stringency increased was supported by six other studies [16,38,44,52,59,305] across the world (e.g. 37 OECD countries, 30 Asian countries and 50 states in the US) during varying periods in 2020. Five studies only found a significant impact on SARS-CoV-2 transmission when the mass gathering restrictions reached the maximum limit of 10 people or fewer [16,35,45,310,311]. The effectiveness of restrictions on mass gatherings also seemed to increase over time. For instance, one study estimated that the restriction of public gatherings of more than 10 people was associated with a reduction in Rt by 6% on day 7, 13% on day 14 and 29% on day 28 across 131 countries in the first half of 2020 [35].A similar pattern of effect on daily cases was also found in two other studies [28,34]. However, the period of assessment was short (the first half of 2020) and therefore did not consider the long-term implementation of such restrictions, where adherence could have waned in subsequent waves.

## 4. Discussion

This review identified 338 studies that assessed the impact of SDMs on reducing the transmission of SARS-CoV-2 in community settings. Nearly half of the studies included in this review estimated the effectiveness of stay-at-home orders, 79% of which were found to substantially reduce transmission. The main response variable assessed was the Rt of SARS-CoV-2 in national and multi-national settings. As the effectiveness of interventions differed across populations and across demographics within a population, as well as potentially over time, examining the experiences in multiple countries was beneficial to understand the average effect and variations across the world. Further research could examine the drivers of policy impact, such as the degree to which human behaviour changed, especially across time, as most studies for the stay-at-home orders review were carried out in 2020. The potential for interactions with individual protective measures is also worthy of further study. Logically, as contacts are reduced, the Rt of SARS-CoV-2 becomes smaller, which should result in a decline in transmission and, by extension, a decline in COVID-19-associated morbidity and mortality. While in the minority, nine studies found no significant associations between stay-at-home orders and morbidity and mortality, suggesting that other voluntary behavioural changes had occurred before more stringent interventions were implemented. This highlights one of the key considerations for SDM policies, namely that a policy will only have an effect if it changes the behaviours or actions of individuals in the community. While we reviewed the impact of various SDMs, most studies did not have quantitative data on the degree to which behavioural changes occurred in response to the SDMs.

Estimating the effects of individual interventions proved challenging. By 30 May 2020, five SDMs (closures of schools, workplaces and public transport, restrictions on mass gatherings and public events and restrictions on movement) were already implemented in 118 countries out of 149 countries examined [29]. Therefore, a common difficulty for the studies included in this review was disentangling the effects of any one of the SDMs from other interventions applied at the same time or further understanding the incremental benefit of each. High-quality randomized controlled trials are also infeasible for many SDMs and as such observational evidence and modelling studies guided decision makers on appropriate measures. With that said, studies have made use of different statistical models to reliably estimate the effects of individual measures indicating that the more stringent interventions such as stay-at-home orders followed by restrictions on mass gatherings and school closures were likely to be the most effective at reducing the transmission of SARS-CoV-2. Thus, the combined effects of numerous SDMs, including school closures, workplace closures and restricting mass gatherings were highly successful at reducing transmission in the community. This was also evident by the lack of other respiratory viruses circulating during the COVID-19 pandemic and the subsequent increase in influenza and respiratory syncytial virus transmission upon relaxation of all non-pharmaceutical interventions [170,313–315].

Human mobility became a common indicator for estimating the impact of non-pharmaceutical interventions. Mobility data can arise from public transportation data or aggregated mobile device data to estimate the movements and mixing of a population. When SDMs are implemented, we would expect to see corresponding reductions in mobility and, therefore, contacts. Eighteen studies [13,17,18,27,30,36,55,188,198,210,219,234,237,240,245,246,248,283] included in this review used human mobility as a proxy for estimating the effect of interventions, although we did not explicitly include human mobility in the search terms of our review. Searching all abstracts that were reviewed for the term ‘mobility’ yielded 267 results. While some may not have been relevant to our outcomes, because mobility was not included as a search term, it is likely that many have been missed. Sixteen of the eighteen studies used Google mobility data (mobile phone devices), which has been shown to accurately explain the transmission of SARS-CoV-2 [316–319]. Mobility data could also be used to assess the level of adherence to interventions in place. Adherence, or changes in a population's behaviour, can impact the effectiveness of SDMs. Unintended changes in mobility and population behaviour can be prompted by the initial measures put in place. For example, when schools close, some parents may require work-from-home arrangements, reducing the potential for transmission in the workplace.

Stringent community-wide measures come at a high cost to society. However, studies that examined unintended consequences of non-pharmaceutical interventions, such as mental health, were not included in the scope of this review. Nonetheless, we recognize that due to unintended consequences of school and workplace closures, measures to reduce person-to-person contact and thereby reduce the risk of SARS-CoV-2 transmission within both settings may be preferred to substantially limit viral transmission while limiting the adverse educational and socioeconomic effects. More evidence is needed to identify the optimal combination of measures to be implemented in these settings. Most studies in this review were population-based studies. While observational evidence has its limitations, it will remain an important source of information to guide decision-making in future epidemics or pandemics. Subgroup analyses were not frequently assessed and may be a focus for future research. Overall, countries adopted different approaches to managing COVID-19, and many of the SDMs were implemented together. Even though different SDMs may have varied acceptability and feasibility in different socioeconomic and cultural settings, the studies included in this review suggested that the combination of measures was successful in slowing or even stopping the spread of COVID-19, even though some individual effects and optimal combinations are unclear. When considering SDMs in future pandemics, the potential effectiveness of individual or combination of SDMs needs to be assessed in the context of pathogen transmission dynamics and balanced against the socioeconomic impacts of such interventions.

## Data Availability

The data are provided in the electronic supplementary material [320].
